# Propagation characteristics and prediction of airblast overpressure outside tunnel: a case study

**DOI:** 10.1038/s41598-022-24917-9

**Published:** 2022-11-29

**Authors:** Xianshun Zhou, Xuemin Zhang, Lichuan Wang, Han Feng, Chenzhi Cai, Xiaohui Zeng, Xuefeng Ou

**Affiliations:** 1 School of Civil Engineering, Central South University, Changsha, 410075 China; 2 Key Laboratory of Heavy-haul Railway Engineering Structure, Ministry of Education, Central South University, Changsha, 410075 China; 3 China Railway 18Th Bureau Group Co., Ltd, Tianjin, 300222 China; 4 School of Safety Engineering and Emergency Management, Shijiazhuang Tiedao University, Shijiazhuang, 050043 China; 5 School of civil engineering, Changsha University of Science & Technology, Changsha, 410114 China

**Keywords:** Civil engineering, Engineering

## Abstract

The drilling and blasting method is widely used in tunnel engineering. The accompanying airblast may damage structures and annoy nearby occupants. The prediction of airblast overpressure (*p*_oa_) outside the tunnel is necessary to improve the safety of blasting works. A study of propagation characteristics of airblasts induced by tunnel blasting was carried out through experimental and numerical studies. The results indicate that the distributions of the *p*_oa_ outside the tunnel were anisotropic, which does not conform to the decay law of an explosion in free-field. The propagation of airblasts induced by tunnel blasting is related to the airblast shape. The phenomenon that the *p*_oa_ along the axial direction of the tunnel was higher than along other directions can be explained by the numerical results of the process of airblasts. The airblasts outside the tunnel traveled as a spherical wave, but the pressure was not uniformly distributed. After an airblast plane wave with high speed and high pressure inside the tunnel was transmitted out of the tunnel, its inertia strengthened the pressure in the axial direction. The airblast outside the tunnel is related to the propagation distance *R*_out_, the angle from the measurement to the tunnel axis *α*, and the pressure intensity *p*_0_ at the tunnel portal. Subsequently, an ellipsoidal contour curve of the *p*_oa_ outside the tunnel was plotted, and therefore a new prediction equation was validated by numerical results and field data. Finally, the newly proposed methodology guided the blast design.

## Introduction

The drilling and blasting method has been proven to be the most cost-effective technique for rock fragmentation and has been widely used in tunnel construction. However, this method has undesirable effects, including flying rocks, ground vibration, noise, and airblast^1,2^. Blasting works in neighboring communities may be objectionable to affected residents, and the reaction of these communities can be quite strong even when the negative effects are unlikely to cause damage to building structures^3–5^. Among these negative effects, flying rocks is completely avoidable, and the main aversion of residents comes from vibrations and airblasts. The airblast will impact the walls, roof, and windows of nearby structures and may cause squeaking that annoyance to their occupants^6,7^. In severe cases, large airblast overpressure (*p*_oa_) impulses could cause window failure several kilometers away^8^ and induce various types of hearing impairment^9,10^.

Since the airblast induced by blasting works so annoying, it is necessary to study its propagation characteristics. Explosions in free-field conform to the propagation pattern of spherical waves, the *p*_oa_ decreases inversely as the cube of the distance to blast sources, the most commonly used equation was derived by Hendrich^11^. When the explosion occurs inside confined spaces, spherical waves gradually converted to plane waves^12,13^, the semi-empirical equation for the *p*_oa_ inside tunnels is expressed as^14,15^:

1 $$p_{{{\text{oa}}}} = \left( {2900\frac{m \cdot q}{{S \cdot R}} + 730\sqrt {\frac{m \cdot q}{{S \cdot R}}} } \right)\exp \left( { - \frac{n \cdot R}{{2\sqrt {S/\pi } }}} \right),$$

where *P*_oa_ is the overpressure at a measurement point (kPa), *q* is an explosive charge per delay (kg), *R* is the distance from the measurement point to blast sources (m), and *S* is the cross-section of the tunnel (m^2^). As can be seen from the equation, the decay law of airblast is not only related to the coefficient *q*, *R*, and *S*, the parameters *m* and *n* are also considered. In general, the coefficient *m* was taken to be 0.4, and the coefficient *n* varies with the distance *R*^16^.

Although the airblast propagation characteristics inside the tunnel have been known, it is more important to forecast the *p*_oa_ outside the tunnel through theoretical methods to improve the safety of blasting works. The artificial neural network is a reliable prediction methods^17–19^, and the semi-empirical equation icon method plays a more important role^14,15^. The contour curves of equal *p*_oa_ induced by bench blasting have a shape similar to an ‘egg’ curve, longer at the floor level and shorter at the top^6,20^, which indicates that the *p*_oa_ does not spread uniformly along all directions. In the area near the tunnel, Rodriguez et al.^14^, experimentally measured the *p*_oa_ outside a tunnel, the pressure also does not decay uniformly in a circle around the tunnel portal; then, Rodriguez et al.^15^ proposed a method to plot the contour of the *p*_oa_ curve outside the tunnel; Yet, the method can still be improved and optimized, because the decay paraments of the *p*_oa_ are also influenced by factors such as wind speed^21^, air temperature, and the topographic conditions that it propagates through^22^.

Given that it is not possible to measure every blast, a numerical approach may be a more effective research method. Nowadays, the software ANSYS/LS-DYNA has been widely used in kinetic analysis of blasting works^1^. However, there was little reliable experience to guide numerical simulations of airblasts propagating outside the tunnel, particularly in the transmission of airblasts from the inside to the outside of the tunnel. A reliable numerical model will be helpful for future studies of airblasts.

The study of airblast propagation outside the tunnel is necessary, which ensures it is be controlled below a specified pressure level. In the study, the *p*_oa_ of the tunnel blasting-induced airblasts was investigated by field measurements. Based on the measurements, a three-dimensional numerical model was established and proved, which can simulate the process of the airblasts propagation from the inside to the outside. With the help of numerical analysis, the phenomenon that pressure attenuation varies in different directions was explained. Moreover, a new prediction equation for the *p*_oa_ outside the tunnel along any direction was proposed, which guides blast design.

## Field measurements

### Engineering background

The field test site was the Qifengshan tunnel, a high-speed railway tunnel from the Zhengzhou-Wanzhou Railway in Hubei. The tunnel has a length of 5152 m and was constructed using the New Austrian tunneling method (NATM) in two benches. In the range for blasting tests, the rock of the tunnel was intact overall and of weakly weathered granite.

**Figure 1:**
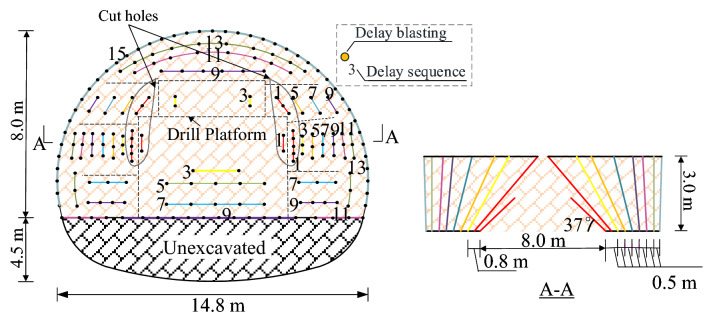
Typical blasting scheme for upper bench blasting.

Figure 1 shows the typical blasting holes scheme and delay sequences. The upper bench height is 8.0 m, and the sectional area is approximately 120 m^2^. The blast is induced by an emulsion explosive, which has high water resistance, and non-electric millisecond delay detonators. The diameters of all the holes are 42 mm, and the depths of the holes range from 1.2 to 3.0 m. The spacing of each blast holes was about 60–80 cm. The cut holes were stemmed with clay.

In the residential area close to the tunnel portal, the airblasts may vibrate the walls, causing dishes to shake and floor to wobble^21^. The relationship between some typical building damage and the *p*_oa_ is summarized in Table 1. The allowed *p*_oa_ for non-operators, as per the Chinese regulation (GB6722-2014)^23^, is 2.0 kPa.

**Table 1:** Typical effects of pressure on building structures^21–23^.

*p*_oa_		Effect
(kPa)	(dB-L)	
20.0	120	Severely damaged conventional structure
6.32	110	General window breakage
**2.0**	**100**	**Safety permissible standards for personnel, GB6722, China**
0.63	90	Some window breakage
0.20	80	Reasonable threshold to prevent glass and plaster damage, USBM
0.06	70	Fall of loose plaster flakes, USBM RI 8485, and WV regulatory limit
0.02	60	Rattling of windows, feelings of annoyance

Significant values are in bold.

### Measurements

**Figure 2:**
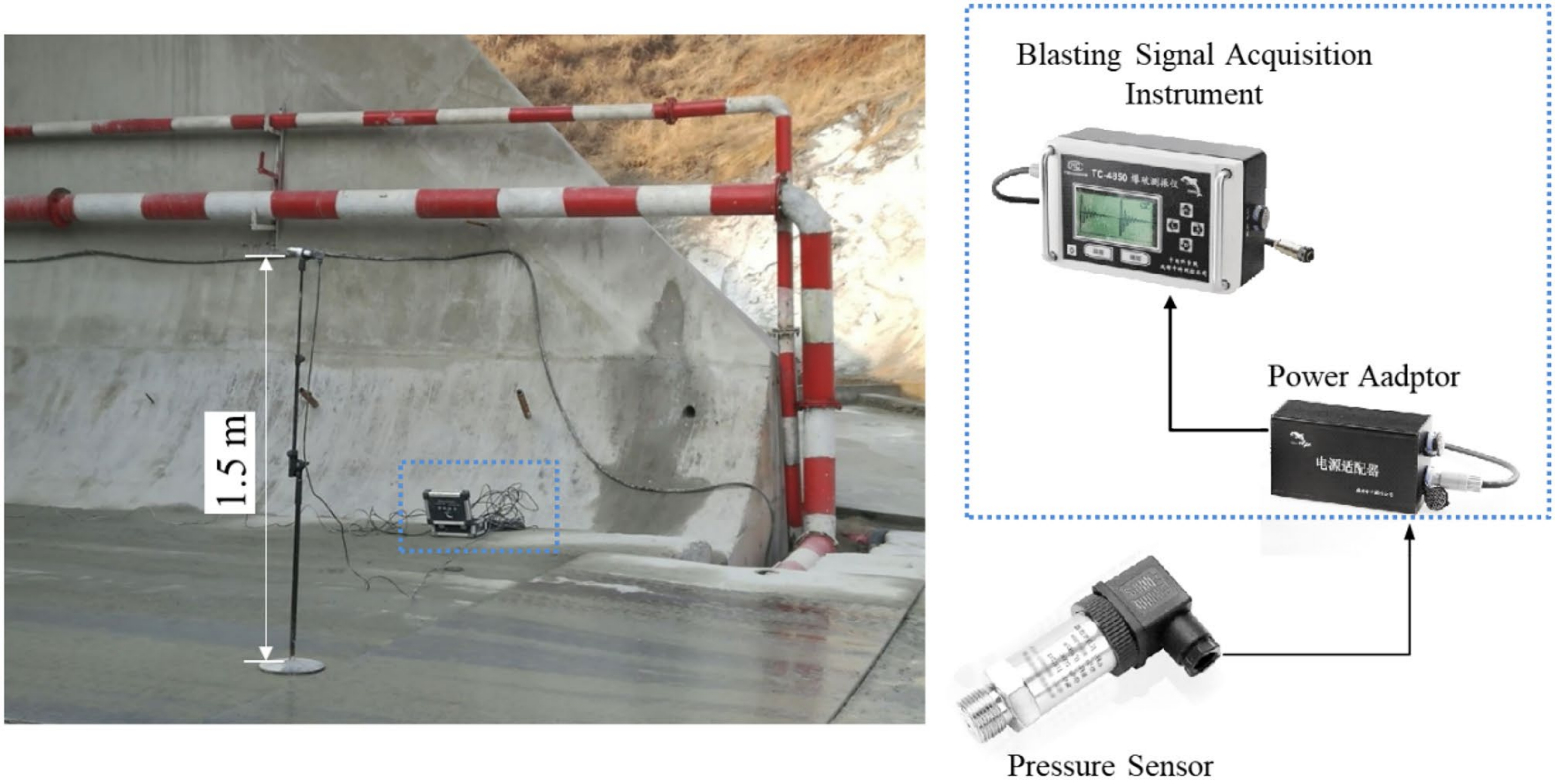
Layout of measurement instruments.

The airblasts were measured by several pressure sensors with the parameters shown in Table 2. The height of the measuring points from the ground was 1.5 m. The layout of the measurement instruments is shown in Fig. 2.

**Table 2:** Parameters of pressure sensors.

Types	Range (kPa)	Sampling frequency (kHz)	Triggered pressure (kPa)	Duration (s)
Pressure sensors	0–250	2.0	0.2	2.0

**Figure 3:**
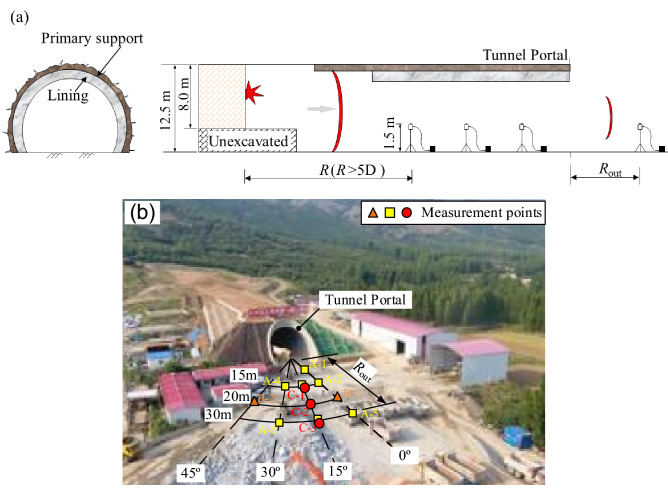
Arrangement of measuring points: (**a**) inside tunnel; (**b**) outside tunnel.

The arrangement of the measuring points is shown in Fig. 3. In the figure, *R* denotes the distance (m) to the blast sources, and *R*_out_ denotes the distance (m) from the measuring points outside the tunnel to the tunnel portal, ranging from 15 to 30 m. The directions *α* (°) between the measurement line and the tunnel axis. We measured a total of eighteen sets of airblasts inside the tunnel and three sets of data at different directions outside the tunnel, the directions α (°) ranging from 0° to 45° (Fig. 3).

## Numerical model

### Configuration

**Figure 4:**
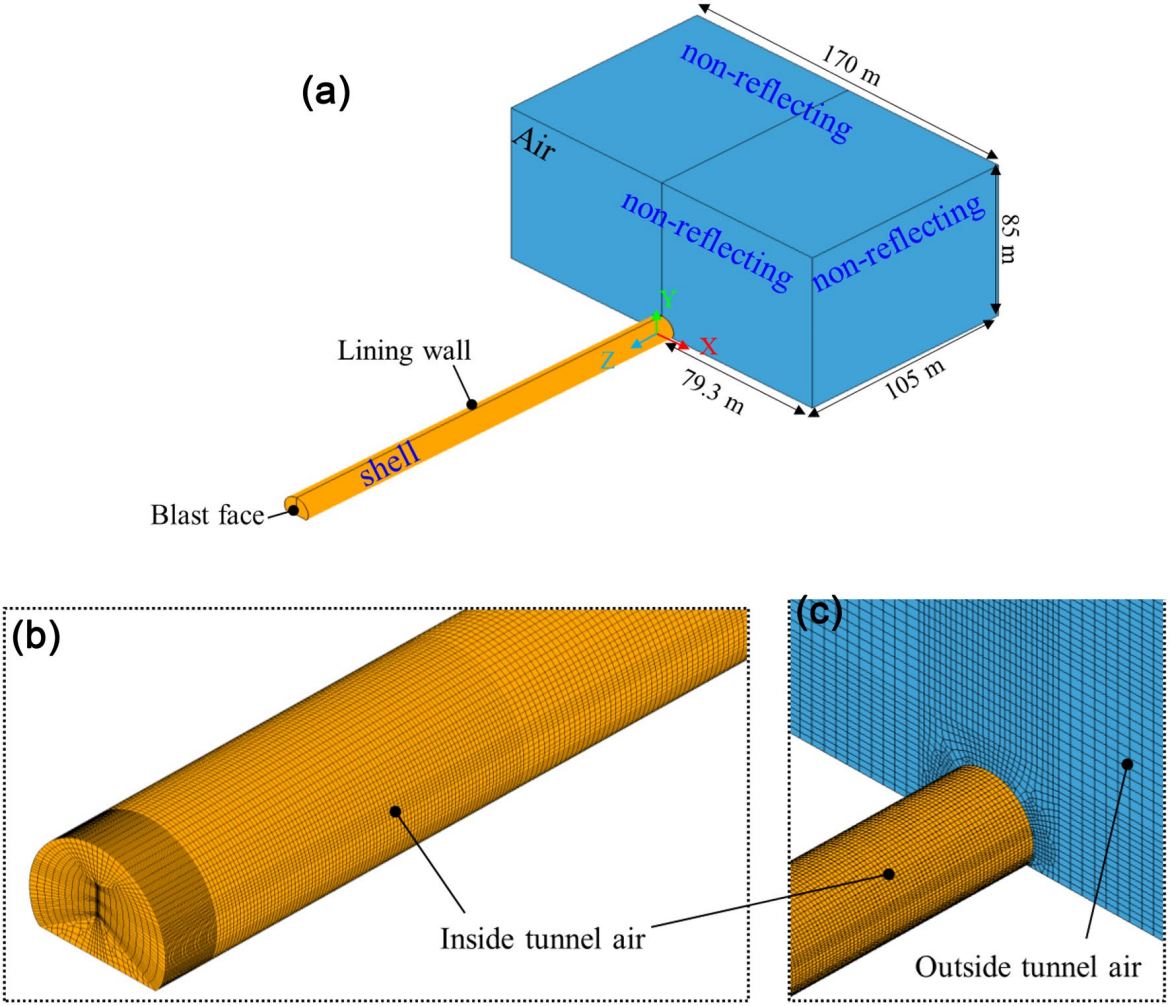
Numerical model: (**a**) model size, (**b**) partial grid near blast face, and (**c**) partial grid near tunnel portal.

**Figure 5:**
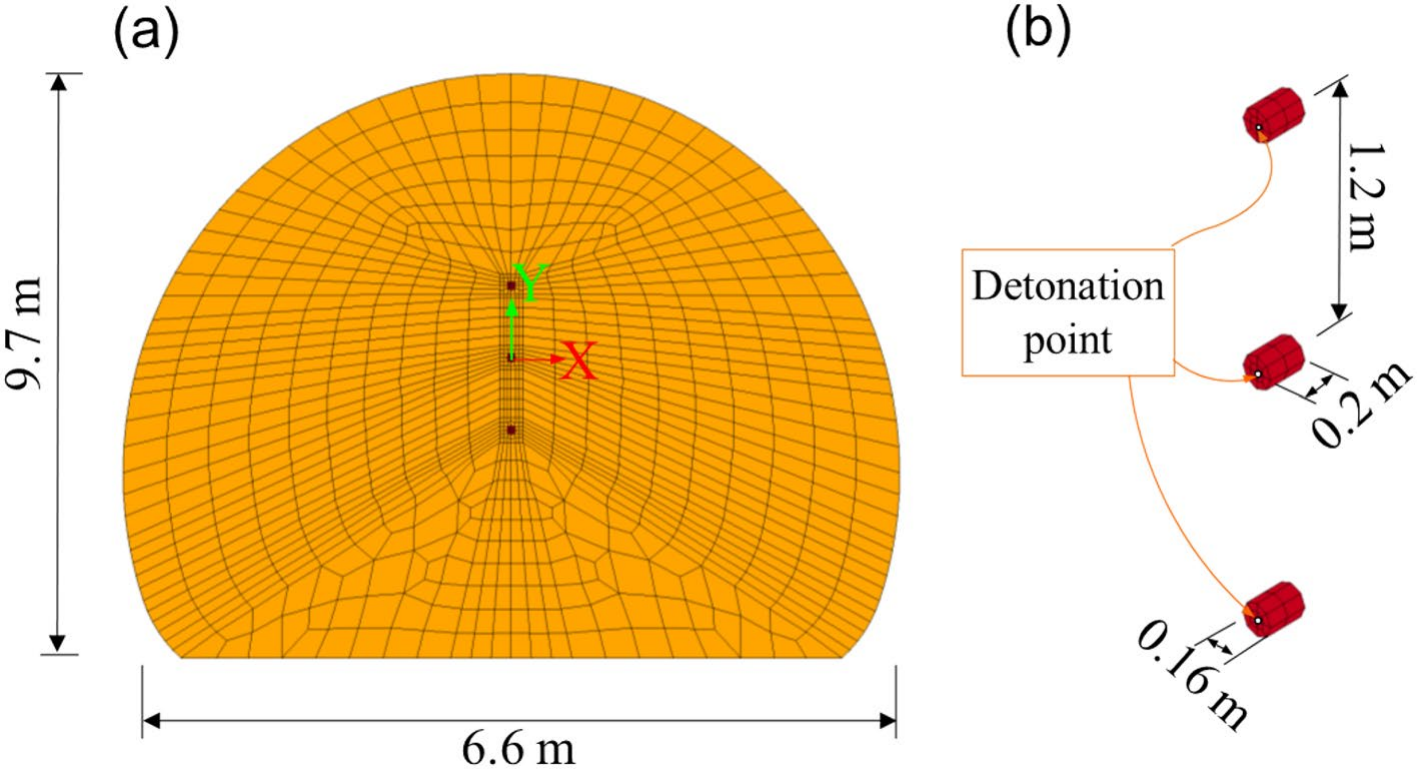
Explosives model: (**a**) tunnel cross-section, and (**b**) explosive charge.

In this study, we used ANSYS/Ls-Dyna to simulate the blasting air wave propagating from inside the tunnel to outside the tunnel. The model size is shown in Figs. 4 and 5. The air size of the outside tunnel was 170 m × 85 m × 105 m (X × Y × Z). The cut holes charge was approximately 48 kg in the field test but only a part of the explosive energy becomes an airblast wave; the energy conversion coefficient was verified to be 0.4^14–16^. In addition, the airblast wave is simulated by detonating the equivalent of the TNT explosion, and the TNT equivalent coefficient of rock emulsion explosive is 0.625. Therefore, to simplify the calculation, the explosive was equivalent to three-cylinder charges, and the charge exposed simultaneously in the numerical calculation was 12 kg. The detonation was performed at the bottom of the hole. The terrain outside the tunnel is flat and symmetry. The airblast propagating in all directions without considering wind and atmospheric inversion. By using the symmetries of tunnel axis in a model, its size be reduced by half or more, making this an efficient method for simulating long-distance airblast. The number of finite elements was 968,217; the nodes of the model were 920,133. The end of the tunnel, as well as the wall and the bottom of the tunnel, are lining which are set as shell elements, constrained by displacement and velocity; and the air outside the tunnel was the non-reflecting boundary.

The Jones-Wilkins-Lee (JWL) equation of state (EoS) is commonly used in explosives modeling for describing the pressure–volume–energy relationship of detonation products^13^. The JWL expression is:

2 $$p = A\left( {1 - \frac{\omega }{{R_{1} V}}} \right)e^{{ - R_{1} V}} + B\left( {1 - \frac{\omega }{{R_{2} V}}} \right)e^{{ - R_{2} V}} + \frac{{\omega E_{0} }}{V},$$

where *ρ* is the density of the explosive, *A*, *B*, *R*_1_, *R*_2_, and *W* are constants determined from experiments, and *E*_0_ is the initial specific internal energy. The relative volume *V* is used to describe the expansion during the explosion, generally taken as 1.0. The calculation parameters are from the experiment^24^, as shown in Table 3.

**Table 3:** Explosive material parameters.

*ρ* (kg·m^-3^)	*E*_0_ (GPa)	*A* (GPa)	*B* (GPa)	*R*_1_	*R*_2_	*w*
1600	3.0	540	9.4	4.5	1.1	0.35

### Validation of model

**Figure 6:**
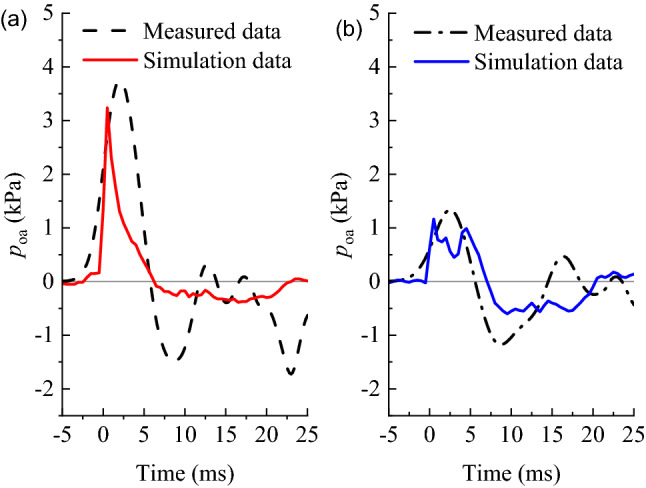
Comparison of simulation and field measurements: (**a**) inside the tunnel; (**b**) outside the tunnel.

Figure 6 depicts the comparison between the *p*_oa_ of history curves at the measuring points inside and outside the tunnel. It can be observed that the numerical simulation curve and the field-measured curve fitted well. Notes: The distance from the measurement points to the tunnel portal are 15 m.

## Results and analysis

### Propagation characteristics

**Figure 7:**
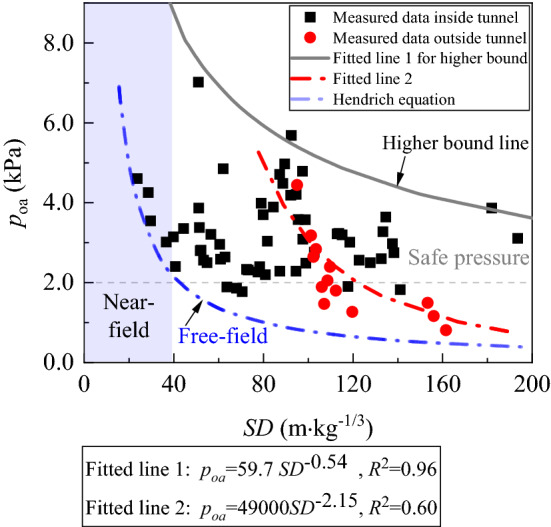
Relationship between the *p*_oa_ and the *SD*.

Figure 7 depicts the airblast measured in the Qifengshan Tunnel. The range of the *p*_oa_ are 1.9–7.0 kPa inside the tunnel and 0.3–4.5 kPa outside the tunnel. The airblasts attenuated rapidly outside the tunnel, while it is slowly inside the tunnel.

Notably, the distribution of the *p*_oa_ inside the tunnel is rather discrete, which is related to the reflection and superposition of the airblast inside the tunnel. The pressure jump caused by the airblasts superposition originates from the conversion from a spherical wave to a plane wave. The airblast advancing inside the tunnel, its pressure is always stronger than the predicted value in the free-field by Hendrich equation^11^.

There is no accurate method for predicting the magnitude of the jump, which is also related to factors such as change of section^25^, wall friction^26^, and large mechanical equipment^27^. Before the airblast converted to a plane wave, the jump also correlated with the size of the propagation space^13^. Cross‑Cracks are naturally present in the rock^28^, and the airblast may rush out of them. The cracks, like the blasting parameters, also affect the intensity of the airblast^29^.

The level of pressure decay differs between the inside and outside tunnel, which may be related to the airblast shape. Mathematical equations with physical connotations are introduced to facilitate the comparison of attenuation levels. In a near-field explosion, the airblast shape is almost like a spherical^11^, and the pressure is proportional to the explosive charge *q* and inversely proportional to the surface of the sphere *S*. This functional relationship can be approximated as:

3 $$p_{oa} \propto \frac{m \cdot q}{S} = \frac{1}{4/3\pi } \times \left( {\frac{{(m \cdot q)^{1/3} }}{R}} \right)^{3} = \frac{1}{4/3\pi } \times SD^{ - 3} ,$$

hence, this relationship describing the cube root of the *q* versus *R* is called the cube-root scaled distance (*SD*). As a result, the relationship between the *p*_oa_ and the *SD* is explained according to the equation:

4 $$p_{\max } = a \cdot SD^{ - b} ,$$

where *a* and *b* are derived from experiments. Moreover, the upper bound of the *p*_oa_ plotted against *SD* in Fig. 7 can be fitted by power equations as follows:

Upper bound (*R*^2^ = 0.96),

5 $$\Delta P = {59}{\text{.7 }}SD^{{ - 0.{54}}} .$$

The pressure on the outer axis outside the tunnel can be predicted as follows (*R*^2^ = 0.60):

6 $$\Delta P = 4.9 \times 10^{4} \, SD^{ - 2.15} .$$

In the region close to the blasting source, the measured airblast data conform to the decay law of the spherical wave. The attenuation curve outside the tunnel is also similar in magnitude to the curve of a free-field explosion. It should also be approximately spherical when the airblast propagates outside the tunnel. Yet, the *p*_oa_ outside the tunnel is higher than that predicted in the free field. The airblast outside the tunnel is related not only to the charge of explosives and the propagation distance but also to the *p*_oa_ at the tunnel portal. The *p*_oa_ at the tunnel portal is not easily attenuated due to the reflection and superposition inside the tunnel.

### Process of airblast conversion

**Figure 8:**
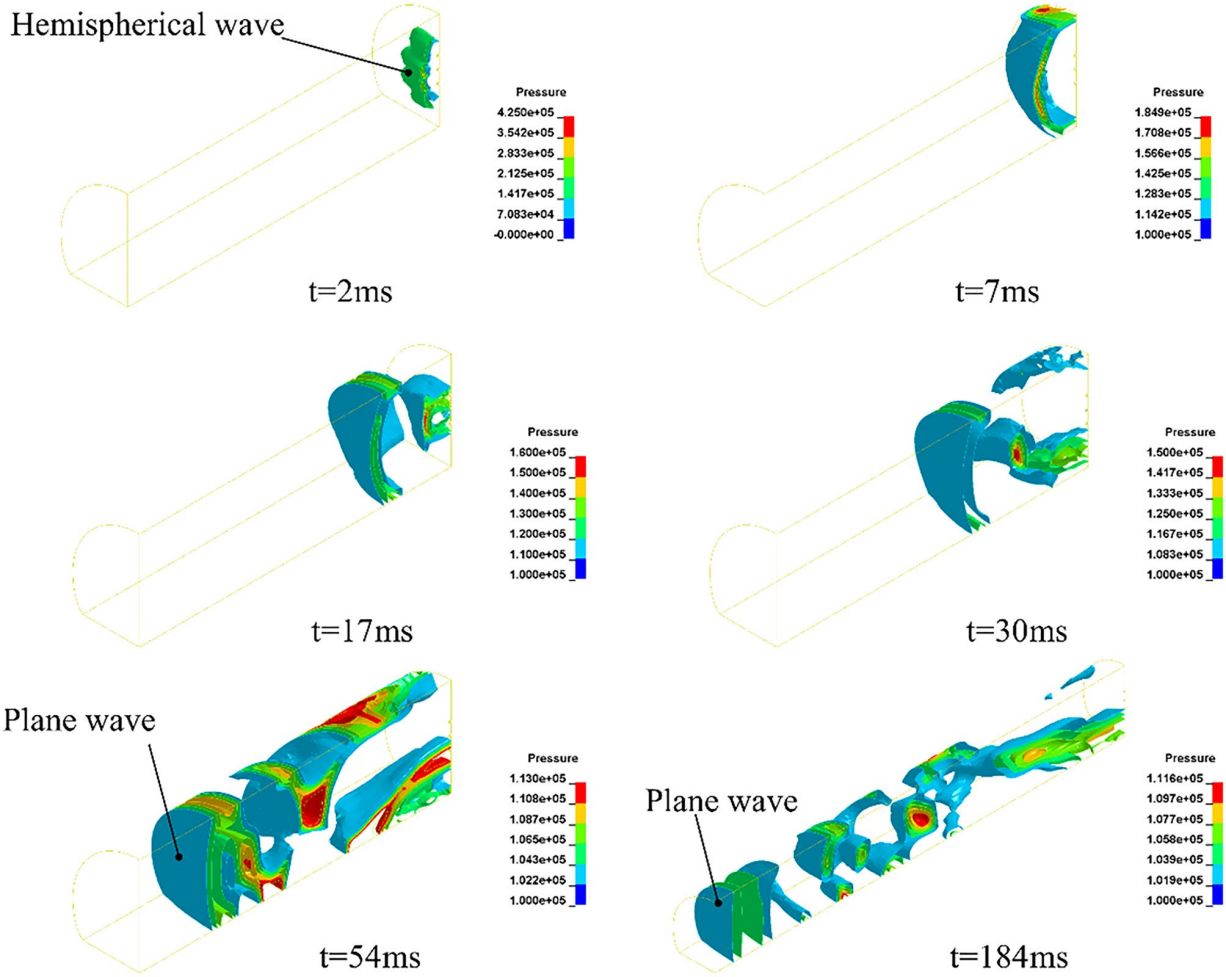
Process of airblast conversion inside the tunnel.

In the analysis of Fig. 7, the pressure decay is closely related to the shape of the airblast; and this phenomenon can be reproduced by numerical simulation. Figure 8 depicts a three-dimensional wavefront of an airblast, from a hemispherical wave to a plane wave*.* The hemispherical wave is caused by three explosives and then reflected from the wall. The reflected wave gradually caught up with the initial wave. After a period of reflection and superposition of distance has occurred, then forms a plane wave at a certain location^13^.

**Figure 9:**
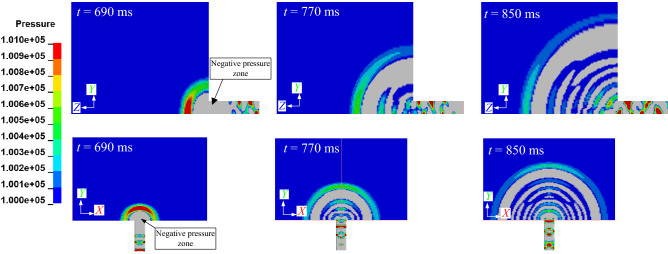
Process of airblast propagation outside the tunnel.

Figure 9 depicts the airblast spreading out in a spherical manner from inside to outside the tunnel. The continuous expansion of the spherical waves leads to a decrease in pressure on the surface. But, distinguishing from the spherical waves in the near field, the pressure at the spherical surfaces is not uniform. The phenomenon of inhomogeneous pressure drop would be verified with the elliptical curve of the measurements *p*_oa_ in Fig. 11.

**Figure 10:**
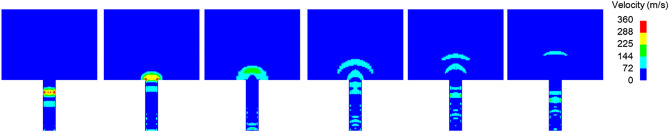
Airblast propagation speed.

The pressure propagation of the airblast is velocity-dependent (Figs. 9 and 10). The shape of the airblast outside the tunnel is nearly spherical. But, its propagation speed varies in different directions. The airblast inside the tunnel is a wave at the speed of sound; at the axis direction outside the tunnel, it possesses the maximum inertia of motion. This high-speed inertia strengthens the pressure in the tunnel axis direction.

### The distribution curve of airblast outside the tunnel

**Figure 11:**
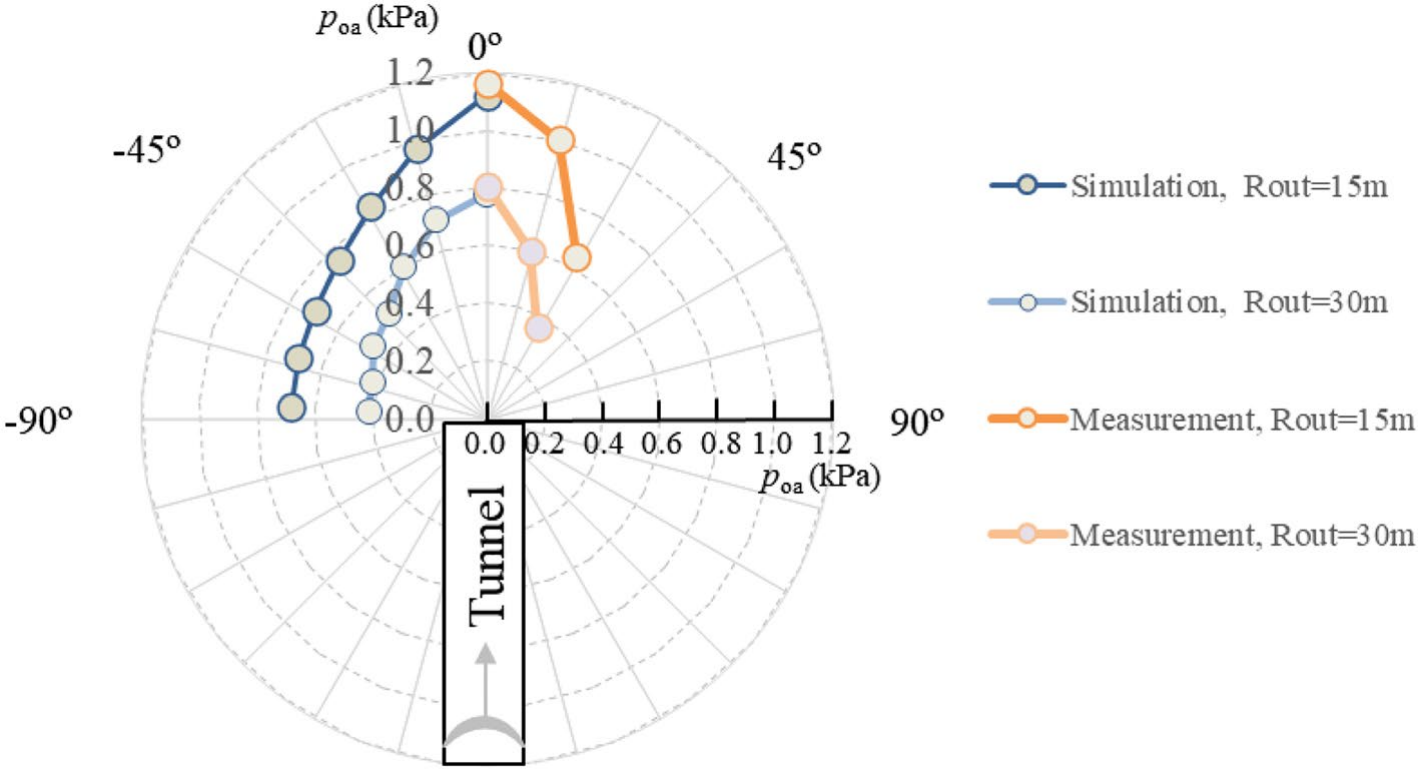
Distribution curve of *p*_oa_ outside the tunnel.

Figure 11 depicts the ellipse-like distribution curve of the *p*_oa_ outside the tunnel. In the figure, the range 0°–90° shows the field measurements, and the range − 90°–0° shows the numerical simulation data.

The airblast attenuation shows an anisotropy in different directions, with a higher intensity along the tunnel axial direction. The *p*_oa_ outside the tunnel is related to the propagation speed of the airblast, which is reproduced in the numerical simulation. As the airblast advanced by an approximate sound speed inside the tunnel, the inertia of this high speed causes a strengthening along the tunnel axis direction. The pressure curve behaves like an ellipse accordingly. The *p*_oa_ at the axis outside the tunnel is inversely proportional to the square of the distance from the source (as in Fig. 7); then, in the other directions outside the axis, the decay law could apply as well, although the decay degree may vary at any direction. Moreover, the shape of the ellipse curves of *p*_oa_ not only related to the degree of attenuation at locations Rout but most importantly the intensity of the elliptical center, the *p*_oa_ of the airblast at the tunnel portal.

## Prediction for airblast

### The *p*_oa_ at the tunnel portal

The *p*_oa_ that the airblast propagated to the tunnel portal is important to predict the *p*_oa_ outside the tunnel. The parameter *n* is the only unknown parameter in Eq. (1). The upper bound used as the predicted value at the tunnel portal ensures that the impact of airblasts would not be underestimated. The relationship is established according to Eqs. (1) and (5):

7 $$59.7\left( {\frac{R}{{(m \cdot q)^{1/3} }}} \right)^{ - 0.54} = \left( {2900\frac{m \cdot q}{{S \cdot R}} + 730\sqrt {\frac{m \cdot q}{{S \cdot R}}} } \right)\exp \left( { - \frac{n \cdot R}{{2\sqrt {S/\pi } }}} \right),$$

after transformation as:

8 $$n = \frac{{2\sqrt {S/\pi } }}{R}\left[ {\ln \left( {2900\frac{m \cdot q}{{S \cdot R}} + 730\sqrt {\frac{m \cdot q}{{S \cdot R}}} } \right) - 0.18\ln \left( {m \cdot q} \right) + 0.54\ln R + 2.2} \right].$$

**Figure 12:**
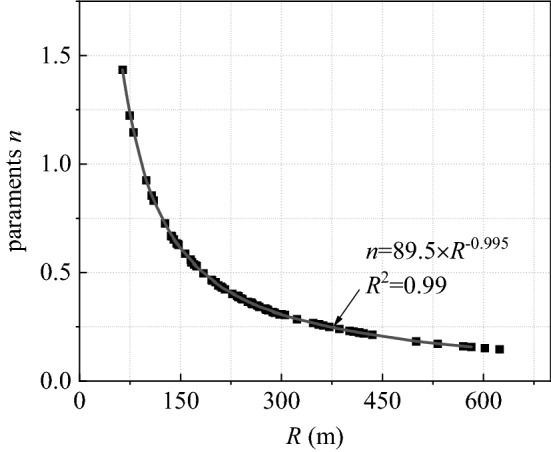
Variation of parameter *n* with *R*.

Figure 12 illustrates the relationship between parameter *n* and the *SD*. For blasting work in the Qifengshan tunnel, parameters *S* and *q* have already been known, and the parameter *n* can be selected by the distance *R* from the predicted location to the blast face.

### Prediction of the *p*_oa_ outside the tunnel

Rodriguez et al.^14,15^ painted the iso-attenuation curves of airblast which requires data from four locations with the *α* = 90, 180, 270, and 360, corresponding to the four directions, parallel and perpendicular, to the tunnel portal. However, the field measurements in this study were not following their requirements. Therefore, we proposed a new prediction equation.

A point acoustic source produces a spherical acoustic wave, the propagation pressure of it with the intensity of the source and the propagation distance^30^. The propagation characteristics are consistent with the decay law of airblast traveled in a spherical wave. The airblast is inversely proportional to the cube of distance; and proportional to the amount of explosive. The airblast propagating outside the tunnel is not exactly a uniform spherical wave; the *p*_oa_ of it is related to the angle. Therefore, an equation is established similar to the point source propagation equation. The equation is related to the *p*_oa_ at the tunnel portal (*p*_0_) and propagation distance outside the tunnel (*R*_out_):

9 $$p_{oa} = \frac{{p_{0} }}{{R_{{{\text{out}}}}^{\eta } }},$$

then, the attenuation coefficient *η* of the airblast at different angles is expressed by the following equation:

10 $$\eta = \frac{{\log_{10} \left( {p_{0} } \right) - \log_{10} \left( {p_{oa} } \right)}}{{\log_{10} \left( {R_{out} } \right)}}.$$

**Figure 13:**
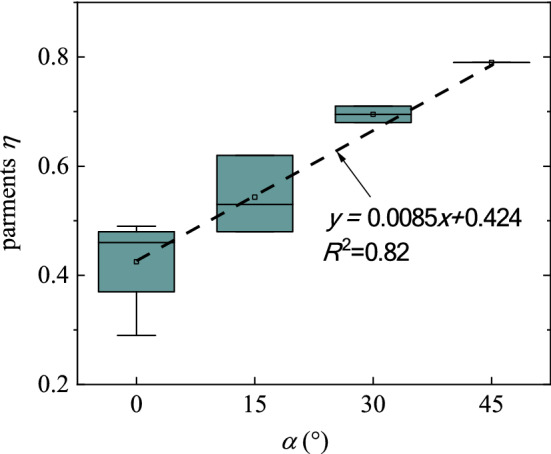
Variation of parameter *η* with *α*.

Table 4 depicts the measurement data of the airblast outside the tunnel. The distribution curve of the airblast is similar to an ellipse (as Fig. 11), which indicates that the airblast does not attenuate uniformly in all directions. The relationship between the attenuation coefficient *η* and the angle *α* can be calculated by Eq. (10), as shown in Fig. 13. The attenuation coefficient *η* is linearly related to the angle *α*.

**Table 4:** Data in field measurements.

No	*q* (kg)	*R* (m)	*R*_out_ (m)	*p*_oa_ (kPa)	*p*_0_ (kPa)	*α* (º)	*η*
A-0	51.6	413	0	–	3.98	0	–
A-1	51.6	413	7.5	1.50	3.98	0	0.49
A-2	51.6	413	15	1.16	3.98	0	0.45
A-3	51.6	413	15	1.01	3.98	15	0.51
A-4	51.6	413	15	0.64	3.98	30	0.68
A-3	51.6	413	30	0.81	3.98	0	0.47
A-5	51.6	413	30	0.62	3.98	15	0.55
A-6	51.6	413	30	0.36	3.98	30	0.71
B-0	49.6	281	0	–	4.19	0	–
B-1	49.6	281	20	2.06	4.19	0	0.29
B-2	49.6	281	20	0.46	4.19	45	0.79
C-0	58.8	294	0	–	4.90	0	–
C-1	58.8	294	15	1.35	4.90	15	0.48
C-2	58.8	294	20	1.00	4.90	15	0.53
C-3	58.8	294	30	0.60	4.90	15	0.62

## Discussion

**Figure 14:**
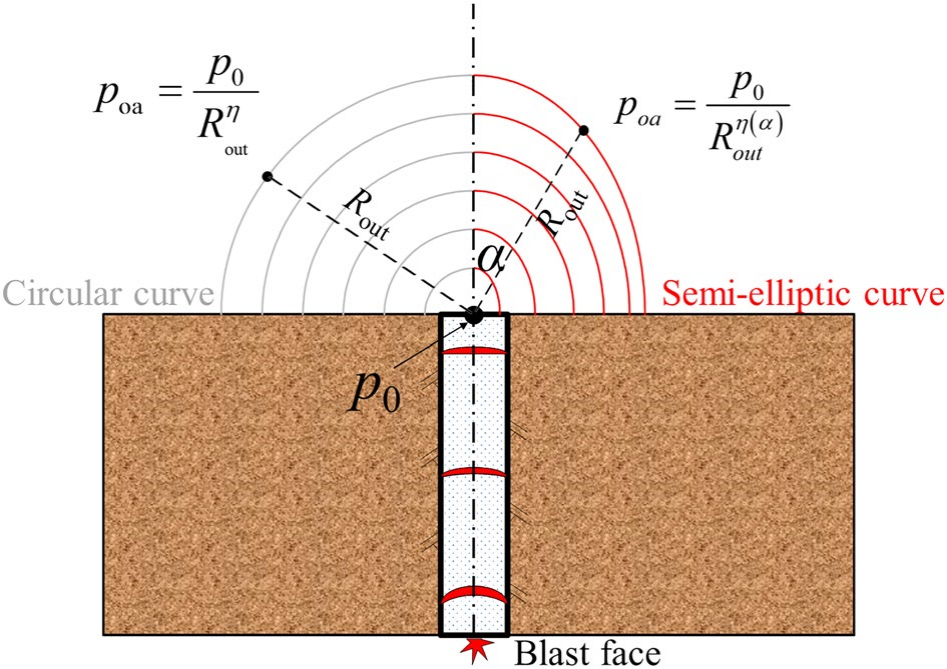
Distribution curve form of airblast outside the tunnel.

The distribution curve of the *p*_oa_ outside the tunnel depicts a semi-ellipse, as shown in Fig. 14. The propagation of the airblast induced by tunnel blasting is related to the airblast shape. In a field near the blast source, the decay law of airblast conforms to a spherical wave (Figs. 7 and 8). The decay degree of pressure is fast. Further, the airblast outside the tunnel also propagated as a spherical wave, and the pressure of it decay fast (Figs. 7 and 8). But, it is different from the near field, the pressure on the airblast spherical wave outside the tunnel is not uniform (Figs. 9 and 11). The airblast decays the slowest along the tunnel axial direction, as the high-speed inertia strengthens the pressure in the axial direction (Figs. 9 and 10). At other directions, the coefficient of attenuation is linearly related to the angle (Fig. 13). Therefore, the airblast outside the tunnel is not only related to the propagation distance *R* and the charge of explosives *q* but more relevant to the pressure intensity *p*_0_ at the tunnel portal. Finally, the angle *α* of measurements to the tunnel axis and the distance *R*_out_ to the portal are needs to be considered in a predicted *p*_oa_ over a wide area.

To summarize, a new equation is established to construct a relationship between *p*_oa_ located at the measuring point and *p*_0_ located at the tunnel portal, in which the attenuation coefficient *η* is related to the directions angle *α* outside the tunnel.

11 $$\left\{ {\begin{array}{*{20}l} p_{oa} = \frac{{p_{0} }}{{R_{out}^{\eta \left( \alpha \right)} }},\eta \left( \alpha \right) = \eta_{axis} + k \times \alpha \, (for \, - 90^{ \circ } < \alpha < 90^{ \circ } ) \\ p_{0} = \left( {2900\frac{m \cdot q}{{S \cdot R}} + 730\sqrt {\frac{m \cdot q}{{S \cdot R}}} } \right)\exp \left( { - \frac{n \cdot R}{{2\sqrt {S/\pi } }}} \right) \\ \end{array} } \right.$$

The coefficient *k* determines the attenuation degree of the coefficient *η* in any direction, and the *η*_axis_ denotes the value at the axial direction of the tunnel. The coefficients are closely related to the terrain of the tunnel site and meteorological conditions.

The prediction method in the study is compared with the Rodríguez methods^14^ in Table 5. As already mentioned, Rodríguez's method needs to measure at least two directions: 0° and 90°; and the predicted values using Rodríguez's method are estimated data. So, his method does not apply to the Qifengshan tunnel. The accuracy is higher with the prediction method in this study, the errors were improved from 54.7 to 3.3%.

**Table 5:** Comparison of predictions and measurements of airblast.

*R*_out_ (m)	*α* (°)	Measured values (kPa)	Predictions					
			*η*	Predicted of this study (kPa)	Errors (%)	*η*	Predicted of Rodríguez^14^ (kPa)	Errors (%)
7.5	0	1.50	0.49	1.48	1.1	0.42	2.77	84.6
15	0	1.16	0.45	1.18	1.4	0.42	1.93	66.1
15	15	1.01	0.51	1.00	1.0	0.48	1.74	72.0
15	30	0.64	0.68	0.63	1.4	0.62	1.36	113.2
30	0	0.81	0.47	0.80	0.7	0.42	0.93	15.2
30	15	0.62	0.55	0.61	1.1	0.48	0.76	22.3
30	30	0.36	0.71	0.36	1.2	0.62	0.47	29.9
20	0	2.06	0.29	1.76	14.7	0.42	1.59	22.7
20	45	0.46	0.79	0.39	14.6	0.77	0.71	54.7
15	15	1.35	0.48	1.34	1.1	0.48	2.14	58.4
20	15	1.00	0.53	1.00	0.1	0.48	1.62	62.3
30	15	0.60	0.62	0.59	0.9	0.48	0.93	55.6
Average					3.3			54.7

**Figure 15:**
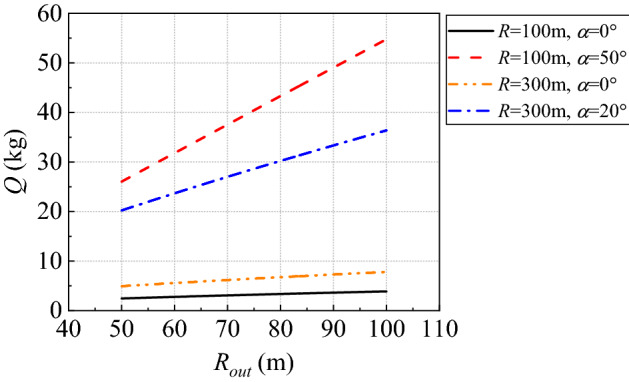
Relationship between *Q* and *R*_out_.

Eventually, we can obtain Eq. (12) for controlling the charge of explosives used in the Qifengshan tunnel based on Eq. (11). This equation shows that, after determining the allowable *p*_oa_ at the measurement point, the charge of explosives *Q* is related to three factors: the propagation distance *R* inside the tunnel, the propagation distance *R*_out_ outside the tunnel, and the angle *α* from the measurement to tunnel axis. This relationship can be obtained from Fig. 15. In the figure, the cross-sectional area of the tunnel is 110 m^2^ and the coefficient *m* is taken as 0.4. The coefficient *n* can then be selected from Fig. 12.

12 $$\left\{ {\begin{array}{*{20}l} Q = 0.0044 \times R\left( {\sqrt {1 + 4.34 \times p_{oa} R_{{_{out} }}^{\eta \left( \alpha \right)} \times \exp \left( {\frac{nR}{{62}}} \right)} - 1} \right)^{2} \\ \eta \left( \alpha \right) = 0.43 + 0.0085 \times \alpha \\ \end{array} } \right.$$

## Conclusion

We comprehensively measured the airblast outside the tunnel, and the propagation characteristics of the airblast were numerically analyzed. The conclusions are summarized as follows:

1. The propagation of the airblast induced by tunnel blasting is related to the airblast shape. The measured *p*_oa_ near the blasting source and outside the tunnel decayed fast, which both conform to the decay law of the spherical wave. Yet, the *p*_oa_ outside the tunnel is higher than that predicted in the free field. A spherical airblast is gradually transformed into a plane wave inside the tunnel, and pressure jumps occur during this process. Then, the pressure decay inside the tunnel becomes slower until it reverts to spherical outside the tunnel.
2. The distributions of the *p*_oa_ outside the tunnel were anisotropic, which does not conform to the decay law of an explosion in free-field. The airblasts outside the tunnel traveled as a spherical wave, but the pressure was not uniformly distributed. At the same distance outside the tunnel, the *p*_oa_ distribution curve has a semi-elliptic shape, longer at the axis direction of the tunnel.
3. The phenomenon that the *p*_oa_ along the axial direction of the tunnel was higher than along other directions is related to the propagation inertia of airblasts. The strengthened pressure along the axial direction is contributed by the high speed and high-pressure airblast inside the tunnel.
4. A new equation predicting the airblast outside the tunnel was proposed. The airblast outside the tunnel is related to the propagation distance *R*_out_, the angle from the measurement to the tunnel axis *α*, and the pressure intensity *p*_0_ at the tunnel portal. The equation fitted well for the field measurements data. Compared to existing methods, the errors were improved from 54.7 to 3.3%. Further, an equation for calculating the charge of explosives under the demand of environmental safe is presented, which is a guideline for the Qifengshan tunnel blasting.

In tunnel blasting engineering, the physical characteristics of the rock are closely related to blasting design, such as rock strength, rock joints, and fissures et al. The influence of these factors on the use of explosive energy and airblast rushes out is also worth studying. The influence of topography, wind speed, and humidity outside the tunnel on the airblast wave propagation is also worth further study. There is still work to optimize the simulation of the influence of large mechanical equipment in the tunnel on airblast wave propagation.

## Data Availability

The datasets generated during and/or analyzed during the current study are not publicly available but are available from the corresponding author at reasonable request.

## References

[1] Jia B, Zhou L, Cui J, Chen H (2021). Attenuation model of tunnel blast vibration velocity based on the influence of free surface. Sci. Rep..

[2] He Z (2021). A combination of expert-based system and advanced decision-tree algorithms to predict air-overpressure resulting from quarry blasting. Nat. Resour. Res..

[3] Faramarzi F, Ebrahimi Farsangi MA, Mansouri H (2014). Simultaneous investigation of blast induced ground vibration and airblast effects on safety level of structures and human in surface blasting. Int. J. Min. Sci. Technol..

[4] Afeni TB, Osasan SK (2009). Assessment of noise and ground vibration induced during blasting operations in an open pit mine—a case study on Ewekoro limestone quarry, Nigeria. Min. Sci. Technol..

[5] Norén-Cosgriff KM, Ramstad N, Neby A, Madshus C (2020). Building damage due to vibration from rock blasting. Soil Dyn. Earthq. Eng..

[6] Segarra P, Domingo JF, López LM, Sanchidrián JA, Ortega MF (2010). Prediction of near field overpressure from quarry blasting. Appl. Acoust..

[7] Richards AB (2010). Elliptical blasting air overpressure model. Min. Technol..

[8] Monk S, Clubley SK (2021). Experimental testing of structural arrangement on window response to the long-duration blast. Eng. Fail. Anal..

[9] Wang Y, Urioste RT, Wei Y, Wilder DM, Arun P, Sajja V, Gist ID, Fitzgerald TS, Chang W, Kelley MW, Long JB (2020). Blast-induced hearing impairment in rats is associated with structural and molecular changes of the inner ear. Sci. Rep.

[10] Kimura E, Mizutari K, Kurioka T, Kawauchi S, Satoh Y, Sato S, Shiotani A (2021). Effect of shock wave power spectrum on the inner ear pathophysiology in blast-induced hearing loss. Sci. Rep..

[11] Henrych J, Abrahamson GR (1980). The dynamics of explosion and its use. J. Appl. Mech..

[12] Silvestrini M, Genova B, Trujillo FJL (2009). Energy concentration factor. A simple concept for the prediction of blast propagation in partially confined geometries. J. Loss Prevent. Proc..

[13] Benselama AM, William-Louis MJP, Monnoyer F, Proust C (2010). A numerical study of the evolution of the blast wave shape in tunnels. J. Hazard. Mater..

[14] Rodríguez R, Toraño J, Menéndez M (2007). Prediction of the blasting air wave effects near a tunnel advanced by drilling and blasting. Tunn. Undergr. Space Technol..

[15] Rodríguez R, Lombardía C, Torno S (2010). Prediction of the air wave due to blasting inside tunnels: Approximation to a ‘phonometric curve’. Tunn. Undergr. Space. Technol..

[16] Fang Y, Zou Y, Zhou J, Yao Z, Lei S, Yang W (2019). Field tests on the attenuation characteristics of the blast air waves in a long road tunnel: A case study. Shock Vib..

[17] Aminshokravi A, Eskandar H, Derakhsh AM, Rad HN, Ghanadi A (2018). The potential application of particle swarm optimization algorithm for forecasting the air-overpressure induced by mine blasting. Eng. Comput..

[18] Hajihassani M, JahedArmaghani D, Sohaei H, Tonnizam Mohamad E, Marto A (2014). Prediction of blasting air-overpressure induced by blasting using a hybrid artificial neural network and particle swarm optimization. Appl. Acoust..

[19] Chen W, Hasanipanah M, Nikafshan Rad H, JahedArmaghani D, Tahir MM (2021). A new design of evolutionary hybrid optimization of SVR model in predicting the blast-induced ground vibration. Eng. Comput..

[20] Jaroonpattanapong P, Tachom K (2021). Monitoring and control blasting air overpressures in an open pit coal mine. Phys. Chem. Earth Parts A/B/C.

[21] Ratcliff J, Sheehan E, Carte K (2011). Predictability of Blasting Air at Surface Coal Mines in West Virginia.

[22] Rustan A, Cunningham C, Fourney W, Spathis A, Simha K (2010). Mining and Rock Construction Technology Desk Reference: Rock Mechanics.

[23] National Standard Writing Group of the People’s Republic of China (2014). Blasting Safety Regulations: GB 6722–2014.

[24] Gao XN, Wu YJ (2015). Numerical calculation and influence parameters for TNT explosion. Chin. J. Explos. Propellants..

[25] Pennetier O, William-Louis M, Langlet A (2015). Numerical and reduced-scale experimental investigation of blast wave shape in underground transportation infrastructure. Process Saf. Environ..

[26] Smith PD, Vismeg P, Teo LC, Tingey L (1998). Blast wave transmission along rough-walled tunnels. Int. J. Impact Eng..

[27] Zhang XM, Zhou XS, Wang LC, Yang GF, Feng H, Gao X, Ma MZ (2020). Attenuation of blast wave in a large-section tunnel. Explos. Shock Waves..

[28] Pour AE, Afrazi M, Golshani A (2022). Experimental study of the effect of length and angle of cross-cracks on tensile strength of rock-like material. Iran. J. Sci. Technol. Trans. Civ. Eng..

[29] Rosenthal, M. F., Morlock, & G. L. Blasting Guidance Manual. Office of Surface Mining 336 Reclamation and Enforcement, US Department of the the Interior (1987).

[30] Zheng CJ (1988). Environmental Noise Control Engineering.

